# Organosilicon-Containing Thiazole Derivatives as Potential Lipoxygenase Inhibitors and Anti-Inflammatory Agents

**DOI:** 10.1155/2007/92145

**Published:** 2007-08-06

**Authors:** Athina Geronikaki, Dimitra Hadjipavlou-Litina, Alla Zablotskaya, Izolda Segal

**Affiliations:** ^1^Department of Pharmaceutical Chemistry, School of Pharmacy, Aristotle University of Thessaloniki, 54124 Thessaloniki, Greece; ^2^Laboratory of Organometallic Chemistry, Latvian Institute of Organic Synthesis, Latvian Academy of Sciences, 21 Aizkraukles Street, 1006 Riga, Latvia

## Abstract

A number of trimethylsiloxyalkyl and trialkylsilylalkyl thiazole derivatives have been evaluated for their anti-inflammatory activity, lipoxygenase inhibiting properties, and cytotoxicity. The investigated compounds have been found to protect in vivo against carrageenin-induced edema, especially 3-(4-trimethylsiloxypiperidin-1-yl)-N-(thiazol-2-yl)-propionamide (**21**) and 2-amino-3-(*γ*-trimethylsilylpropyl)thiazolium iodide (**22**), which exhibited good anti-inflammatory activity: 57.2% CPE inhibition in dose of 0.2 mmol/kg for compound **21** and 55.0% in dose of 0.01 mmol/kg for compound **22**. All the compounds tested inhibited soybean lipoxygenase activity. 2-(4-Trimethylsilyloxypiperidin-1-yl)-N-[4-(*p*-methoxyphenyl)-thiazol-2-yl]-acetamide (**19**) was the most potent displaying inhibition against lipoxygenase (ID_50_ = 0.01 mmol). It also possessed moderate cytotoxic effect (LC_50_ = 13 *μ* g/mL, 3 × 10^−8^ mmol/mL) concerning MG-22A cell lines.

## 1. INTRODUCTION

The aim of this investigation was to study anti-inflammatory as well as lipoxygenase inhibitory activities and cytotoxicity of a series of organosilicon-containing thiazole derivatives.

It is well known that thiazolyl derivatives possess anti-inflammatory activity [1–6]. Today requirements demand novel medicinal remedies possessing different degrees of selectivity and specificity depending on their purpose. Process of inflammation often becomes chronic, and the human organism needs drugs therapy support in periods of acute attacks. Therefore, increase of the variety of specific and selective anti-inflammatory remedies is an important task, especially due to its positive influence on the chronic sick rate decrease. Some anticancer drugs as blenoxane, bleomycine, and tiazofurin, containing thiazolyl moiety in their structure, are known as antineoplastics [7]. Besides, several thiazolyl derivatives were found to be potent antitumour agents [7–9]. Since arachidic acid (AA) metabolism results in the generation of mutagens that damage DNA and induce mutations, members of arachidic acid enzymes, especially the lipoxygenase pathway, have been reported to play a significant role in carcinogenesis. Inhibitors of AA metabolism can reverse the production of these metabolites resulting in recruitment of apoptotic cells clearance [10].

Organosilicon compounds attract scientific attention due to some different reasons, especially due to a number of interesting results in the field of their biological action. Modern organosilicon chemistry coincided with the emergence of biomaterials and bioengineering fields fifty years ago. It has been reported that some organosilicon compounds affect the collagen biosynthesis in cartilagenous tissue [11]. New approaches based on the organosilicon modification of the biologically active compounds, especially of compounds containing hydrophilic functional groups, offer the real possibility to improve their pharmacological properties because of easier penetration of modified compounds through lipophilic barriers inside the body [12, 13]. In this paper, we report the biological activity of trimethylsilyl ethers of thiazole derivatives, but the wide possibility for variation of substituents around the silicon atom can lead to more fine selection of perspective compound for the investigations *in vivo*.

## 2. EXPERIMENTAL

### 2.1. Chemistry

^1^H NMR spectra were recorded with a Mercury 200 (Varian) spectrometer using CDCl_3_ as solvent and hexamethyldisiloxane (HMDSO) as internal standard (for unsilylated compounds). Mass spectra under electron impact conditions were recorded on a Hewlett-Packard apparatus (HP-6890, GC with HP5MS, 70 eV). Analytical thin-layer chromatography (TLC) was performed on Macherey-Nagel silica plastic plates, with visualization under UV (254 mm). Column chromatography was performed using Merck silica gel (0.040–0.063 mm). Solvents and reagents were purchased from the following commercial sources: Fluka, Aldrich, Acros. Melting points were determined on a Boetius melting point apparatus and were uncorrected. Elemental analyses were performed on Carlo Erba 1108 elemental analyzer. Elemental analyses (C, H, N, S) for all compounds synthesized were within ±0.4% of theoretical values. The following compounds were synthesized according to literature procedures: 2-chloro-N-(thiazol-2-yl)-acetamide (**1**) [14, 15], 2-chloro-N-(4-phenylthiazol-2-yl)-acetamide (**2**) [14, 15], 2-chloro-N-[4-(p-methoxyphenyl)-thiazol-2-yl]-acetamide (**3**) [14, 15], 2-chloro-N-(4-phenyl-5-tetradecylthiazol-2-yl)-acetamide (**4**) [14, 15], 3-chloro-N-(thiazol-2-yl)-pro-pionamide (**5**) [14, 15], 2-(4-hydroxypiperidin-1-yl)-N-(thiazol-2-yl)-acetnamide (**6**) [6], 2-(4-hydroxypiperidin-1-yl)-N-(4-phenylthiazol-2-yl)-acetamide (**7**) [6], 2-(4-hy-droxypiperidin-1-yl)-N-[4-(p-methoxyphenyl)-thiazol-2- yl]-acetamide (**8**) [6], 2-(4-hydroxypiperidin-1-yl)-N-(4-phenyl-5-tetradecylthiazol-2-yl)-acetamide (**9**) [6], 3-(4-hydroxypiperidin-1-yl)-N-(thiazol-2-yl)-propionamide (**10**) [6], 4-methyl-5-(*β*-hydroxyethyl)-thiazole (**11**) [16], 4-methyl-5-(*β*-trimethylsiloxyethyl)-thiazole (**12**) [17], 2-amino-4-hydroxymethyl-thiazole (**13**) [18], 2-amino-4-trimethylsiloxymethyl-thiazole (**14**) [17], 2-phenyl-4-hy-droxymethyl-thiazole (**15**) [19], 2-phenyl-4-trimethylsilox-ymethyl-thiazole (**16**) [17], 2-(4-trimethylsiloxypiperidin-1-yl)-N-(4-phenylthiazol-2-yl)-acetamide (**18**) [17], 2-(4-trimethylsiloxypiperidin-1-yl)-N-(4-phenyl-5-tetradecylthi-azol-2-yl)-acetamide (**20**) [17], 3-(4-trimethylsiloxypiperi-din-1-yl)-N-(thiazol-2-yl)-propionamide (**21**) [17], and 2-amino-3-(*γ*-trimethylsilylpropyl)thiazolium iodide (**22**) [17].

#### 2.1.1. 2-(4-trimethylsiloxypiperidin-1-yl)-N-(thiazol-2-yl)- acetamide (**17**)

A mixture of 0.25 mmol (60 mg) of compound **6** and 2.5 mL of hexamethyldisilazane in 5 mL of ether was heated for 25 hours until the precipitate was dissolved. The progress of the reaction was monitored by TLC. When the reaction was complete, the solvent and excess of hexamethyldisilazane were removed in vacuum on a rotary evaporator. The residue was purified by column chromatography on silica-gel (eluent - petr. ether : EtOAc = 1 : 2) to give 71 mg (91%) of the compound **17** as oil. After some time, the crystalls were formed, m.p. 103–105°C.

^1^H NMR (200 MHz, CDCl_3_, 25°C, HMDSO), *δ*, ppm: 0.12 (s, 9H, SiMe_3_), 1.71, 2.42 and 2.82 (m+m+m, 4H+2H+2H, CH_2(cycl.)_), 3.22 (s, 2H, COCH_2_N), 3.74 (s, 1H, CH_(cycl.)_O), 6.98 (d, 1H, 5-H), 7.43 (d, 1H, 4-H). GC-MS: M^+^ = 313 (13%); M^+^-15 (CH_3_) = 298 (2%); M^+^-127 (2-thiazolyl-NHCO) = 186 (100%); M^+^-141 (2-thiazolyl-NHCOCH_2_) = 172 (10%); M^+^-186 (CH_2_-N-(piperidyl)OSiMe_3_) = 127 (8%). Element. anal. found, %: C: 49.50; H: 7.45; N: 13.31; S: 9.03. C_13_H_23_N_3_O_2_SSi (MW = 313.497). Calculated, %: C: 49.81; H: 7.39; N: 13.40; S: 8.96.

#### 2.1.2. 2-(4-trimethylsilyloxypiperidin-1-yl)-N-[4-(pmethoxyphenyl)- thiazol-2-yl]-acetamide (**19**)

A mixture of 120 mg (0.34 mmol) of compound **8** and 3 mL of hexamethyldisilazane in 5 mL of ether was heated with stirring for 100 hours until the precipitate was dissolved and the new one was formed. The progress of the reaction was monitored by TLC. When the reaction was complete, the solvent and excess of hexamethyldisilazane were removed in vacuum in a rotary evaporator. The solid was washed with hexane to give 100 mg (84%) of the compound **19**, m.p. 125–127°C.

^1^H NMR (200 MHz, CDCl_3_, 25°C, HMDSO), *δ*, ppm.: 0.12 (s, 9H, SiMe_3_); 2.79, 2.47 and 1.79 (m+m+m, 2H+2H+4H, CH_2(cycl.)_); 3.22 (s, 2H, COCH_2_N), 3.82 (s, 4H, OCH_3_ + CH_(cycl.)_O); 7.01 (s, 1H, 5-H), 6.93 and 7.74 (d + d, 2H + 2H, CH_(arom)_).

Element. anal. found, %: C: 57.14; H: 6.89; N: 10.06; S: 7.62. C_20_H_29_N_3_O_3_SSi (MW = 419,622). Calculated, %: C: 57.25; H: 6.97; N: 10.01; S: 7.64.

### 2.2. Biological assays

#### 2.2.1. Carrageenin-induced mice paw edema inhibition [20]

AKR or A mice (20–30 g, groups of ten) of both sexes were used. Females pregnant were excluded. A single dose of 0.2 mmol/kg body weight of compounds **12**, **16**, **20**, **21** and 0.01 mmol/kg of compound **22** or 0.013 mmol/kg of compound **14** suspended in water with few drops of Tween 80 was administered *intraperitoneally* simultaneously to the *intradermally* injection of 0.05 mL carrageenin in the right hind paw. Indomethacin was used as a standard diluted agent. Inhibition caused by indomethacin was 57.4% in dose 0.1 mmol/kgbw.

#### 2.2.2. Soybean lipoxygenase inhibition [21]

The tested compounds dissolved in DMSO or ethanol (concentrations ranged from 0.1 to 1 mM) were incubated at room temperature with sodium linoleate (0.1 mmol) and 0.2 mL of enzyme solution (250 U/mL in saline). The conversion of sodium linoleate to 13-hydroperoxylinoleic acid at 234 nm was recorded and compared with nordihydroguaretic acid (0.1 mmol - 84%), an appropriate standard inhibitor.

#### 2.2.3. Cytotoxicity

Monolayer tumour cell lines MG-22A (mouse hepatoma), HT-1080 (human fibrosarcoma), and normal mouse fibroblasts (NIH 3T3) were cultivated for 72 hours in DMEM standard medium (Sigma) without an indicator and antibiotics. After the ampoule had thawed, cells from one to four passages were used in three concentrations of test compound: 1, 10 and 100 *μ*g mL^−1^. The control cells and cells with tested compounds in the range of 2–5* 10^4^ cell mL^−1^ concentration (depending on line nature) were placed on separate 96 wells plates. Solutions containing test compounds were diluted and added in wells to give the final concentrations. Control cells were treated in the same manner only in the absence of test compounds. Plates were cultivated for 72 hours. The number of survived cells was determined using crystal violet (CV), 3-(4,5-dimethylthiazol-2-yl)-2,5-diphenyltetrazolinium bromide (MTT), or neutral red (NR) coloration which was assayed by multiscan spectrophotometer. The quantity of alive cells on control plate was taken in calculations for 100% [22, 23]. The LC_50_ was calculated using Graph Pad Prism 3.0 program, *r* < .05. Concentration of NO was determined according to [23].

## 3. RESULTS AND DISCUSSION

**Figure 1:**
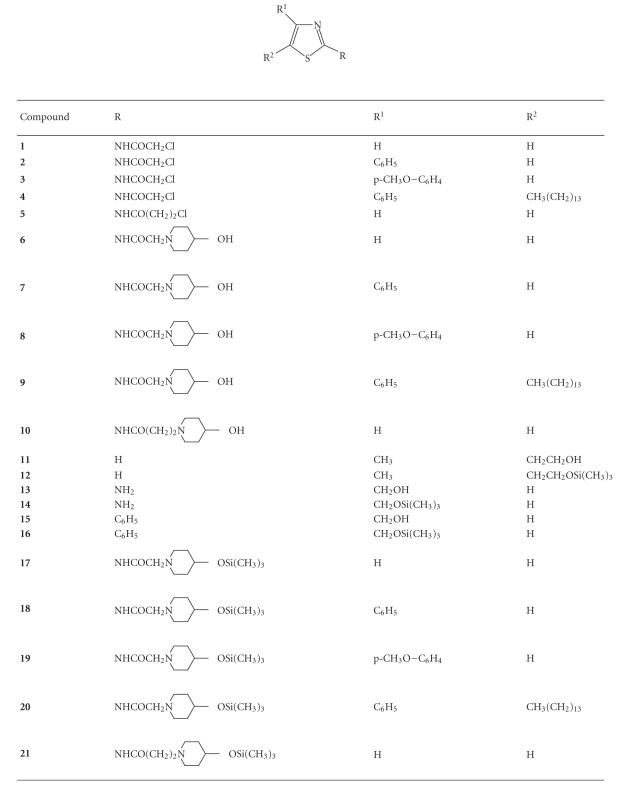
Structure of thiazole derivatives **1**–**21**.

Thiazole derivatives of general formula presented in Figure 1 have been studied.

**Figure 2:**
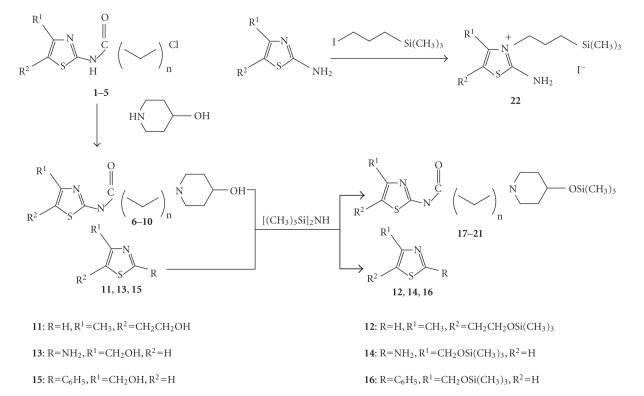
Synthesis of organosilicon derivatives of thiazole.

N-(2-thiazolyl)amides, containing 4-hydroxypiperidine residue, were synthesized by consecutive condensation reactions: 2-aminothiazole reacted with appropriate acyl chloride (chloroacetic or chloropropionic acid chlorides) to give the respective chloroalkylamides (**1–5**) [14, 15], then the reaction of the prepared chloroalkylamides with N-containing heterocycle, 4-hydroxypiperidine, gave the corresponding thiazolyl amides (**6–10**) [6]. The organosilicon derivatives have been prepared in two ways: (a) by introducing of O-silyl group into hydroxyl-containing thiazole compounds, to obtain the compounds **12**, **14**, **16**, **17**–**21**, and (b) by introducing C-silyl group using quarternization reaction of nitrogen to obtain the compound **22** [17]. The general synthetic methods employed are shown in Figure 2.

Structures of the compounds prepared were confirmed by ^1^H-NMR, GC-MS spectroscopy, and by elemental analysis. Theoretical calculations of lipophilicity as clog *P* for compounds synthesized, using the method of additivity, were performed [24] (Table 1). We investigated anti-inflammatory and lipoxygenase inhibitory activities and cytotoxicity of organosilicon-containing thiazole derivatives.

Organosilicon-containing compounds **12**, **14**, **16**, **20–22** were examined *in vivo* for their anti-inflammatory activity using the carrageenin mice paw edema (CPE) as a model of inflammation. The *in vivo* anti-inflammatory effects of the tested thiazole derivatives were assessed by using the functional model of carrageenin-induced rat paw edema and are presented in Table 1 as percentage of weight increase at the right hind paw in comparison to the uninjected left hind paw.

Carrageenin-induced edema is a nonspecific inflammation resulting from a complex of diverse mediators [2]. Since edemas of this type are highly sensitive to nonsteroidal anti-inflammatory drugs (NSAIDs), carrageenin has been accepted as a useful agent for studying new anti-inflammatory drugs [25]. This model reliably predicts anti-inflammatory efficacy of the NSAIDs, and during the second phase it detects compounds which are anti-inflammatory agents as a result of inhibition of prostaglandin amplification.

The studied compounds **12**, **14**, **16**, **20**–**22** were found to protect *in vivo* against edema formation. Analyzing the data obtained, it is revealed that **21** and **22** were more potent among all the compounds tested. Compound **21** exhibited similar to indomethacin inhibition—57.2%, but in double dose (0.2 mmol/kgbw). Organosilicon salt **22** was found to be the most potent inhibitor, possessing about the same as indomethacin inhibition (55.0%), but in lower dose (0.01 mmol/kgbw). 4,5-disubstitued thiazole without 2-substituent (**12**) was found to be the least active compound.

The compounds **12**, **14**, **16**–**20**, and **22** were evaluated for inhibition of soybean lipoxygenase (LOX) by the UV-absorbance-based enzyme assay [26]. While one may not extrapolate the quantitative results of this assay to the inhibition of mammalian 5-LOX, it has been shown that inhibition of plant lipoxygenase activity by NSAIDs is qualitatively similar to their inhibition of the rat mast cell lipoxygenase and may be used as a simple qualitative screen for such activity. The results are presented in Table 1. All the tested compounds were found to inhibit soybean lipoxygenase. The IC_50_ values for compounds **14**, **16**, **17**, **19**, and **22** were determined. They ranged within 0.01–0.47 mmol. For other compounds (**12**, **18**, and **20**) persentage of inhibition at concentration 0.1 mmol was determined.

It has been revealed that among trimethylsiloxyalkyl/trimethylsilylalkyl thiazole derivatives (**12**, **14**, **16**, and **22**), compound **14**, containing 2-amino group, was the most active as lipoxygenase inhibitor (IC_50_ = 0.1 mmol), but **12** without substituent at C_2_-position of thiazole cycle was found to be the least active compound in this respect. It inhibits lipoxygenaze action only by 9.1% in dose of 0.1 mmol.

It was found that among organosilicon-containing 2-thiazolyl-amides **17**–**20**, the presence of substituent in C_4_-position of thiazole ring is essential for lipoxygenase inhibition display. Compounds **19** and **18** were the most potent lipoxygenase inhibitors (IC_50_ = 0.01 mmol, and 66.7% inhibition in dose of 0.1 mmol, correspondingly). Compound **19** was the most active lipoxygenaze inhibitor also among all compounds tested. It was also revealed that the nature of C_4_-substituent influences the degree of inhibition: 4-methoxyphenyl derivative (**19**) was a better inhibitor in comparison with its 4-phenyl analog (**18**). Introduction of additional bulky substituent in C_5_-position of the molecule was telling on the level of inhibition. Thus, compound **20** possessed lower inhibiting properties (by 26%) in comparison with C_5_-unsubstituted compound **18**. Compound **17** without substituent at C_4_-position of thiazole ring was the least potent inhibitor (IC_50_ = 0.35 mmol). Concerning the correlation of lipophilicity—CPE and lipoxygenase inhibition—it was revealed that these parameters do not proceed in parallel along the compounds investigated.

The experimental evaluation of cytotoxicity of compounds **6**, **8**, **17**, and **19** is presented in Table 2.

Compound **8** and its trimethylsilyl ether **19** possess low cytotoxic effect on human fibrosarcoma HT-1080 (LC_50_ > 100 *μ*g/mL) and moderate effect on mouse hepatoma MG-22A (LC_50_ = 17 and 21 *μ*g/mL, correspondingly, CV, and LC_50_ = 16 and 13 *μ*g/mL, correspondingly, MTT coloration). Compound **6** and its trimethilsilyl ether **17** without substituents at C_4_- and C_5_-positions of thiazole do not exhibit cytotoxic properties. Both compounds decrease MG-22A cell growing by up to 40% (MTT coloration), but at the same time, stimulated HT-1080 cell growing at all studied concentrations by up to 55% (CV). No significant difference among compounds was determined comparing their NO-generation ability in HT-1080 cell lines. Compound **19** possessed the highest NO-generation activity concerning MG-22A tumour cells. All studied compounds were nontoxic compounds concerning normal cells NIH 3T3.

Analyzing the results obtained for 2-thiazolyl amides **6**, **8**, **17**, **19** and previously published data on cytotoxicity of silylated compounds **18**, **20**, **21** and their nonsilylated precursors **7**, **9**, **10** [17], it was revealed that all 4-trimethylsiloxypiperidine derivatives of 2-thiazolyl amides **17**–**21** possessed low or moderate cytotoxic effect concerning either human fibrosarcoma (LC_50_ = 44–77 *μ*g/mL) or mouse hepatoma (LC_50_ = 13–59 *μ*g/mL), excluding compound **17**, which was inactive in both tests. The strongest cytotoxic effect on mouse hepatoma was observed for **18** (LC_50_ = 5.3 *μ*g/mL, CV test). It can be noted that all 2-thiazolyl amides, which have bulky substituent in C_4_-position of thiazole ring (compounds **7**–**9** and **18**–**20**), exhibited moderate effect on MG-22A cell line (LC_50_ = 5.3–37 *μ*g/mL, CV). Among unsilylated compounds **7**–**9**, the most lipophilic **9** (clog *P* = 9.134) with additional tetradecyl substituent in C_5_-position possesses the highest cytotoxicity on MG-22A cell line (LC_50_ = 8 *μ*g/mL, CV).

The distance elongation between thiazolyl and piperidyl heterocycles, either in unsilylated compounds **6**, **10** or silylated ones **17**, **21**, leads to cytotoxic effect appearance for propionamides **10** and **21**, concerning human fibrosarcoma (LC_50_ = 48 *μ*g/mL and 44 *μ*g/mL, correspondingly) or its essential increase, concerning mouse hepatoma (LC_50_ = 35 *μ*g/mL for **10** and 44 *μ*g/mL for **21**, CV), in comparison with the corresponding acetamides **6** and **17**.

The introduction of trimethylsilyl group into compound **7** caused the cytotoxic effect increase concerning mouse hepatoma, which was revealed as the highest for **18** (LC_50_= 5.3 *μ*g/mL), among all the compounds studied.

## 4. CONCLUSIONS

The organosilicon thiazoles studied were found to a certain extent to protect *in vivo* against edema formation and to inhibit soybean lipoxygenase. Organosilicon salt **22** was the most potent as anti-inflammatory agent among all compounds tested and indomethacin.

The nature of substituent in C_4_-position of thiazole ring is essentially telling on the degree of lipoxygenase inhibition and cytotoxic activity. The most active as lipoxygenase inhibitor was 2-(4-trimethylsilyloxypiperidin-1-yl)-N-[4-(p-methoxyphenyl)-thiazol-2-yl]-acetamide (**19**), which contains bulky *p*-MeO-C_6_H_4_-group at C_4_-position.

The distance elongation between thiazolyl and piperidyl heterocycles either in parent compounds (**6**, **10**) or their silyl ethers (**17**, **21**) leads to cytotoxic effect noticeable increase for propionamides **10** and **21** in comparison with the corresponding acetamides **6** and **17**. Trimethylsilyl ether **18** was the most active against mouse hepatoma among all the compounds studied and in comparison with its unsilylated precursor **7**.

It can be noted that the data obtained do not allow to conclude definitely the existence of relationship among anti-inflammatory activity, lipoxygenase inhibition, and cytotoxicity. But in some cases, cytotoxic properties were accompanied by anti-inflammatory activity (organosilicon salt **22**) or lipoxygenase inhibition activity display (thiazolyl acetamides **18** and **19**). At the same time, compound 4-methyl-5-(*β*-trimethylsiloxyethyl)-thiazole (**12**) was the least active concerning all the biological properties studied.

The wide possibility for variation of substituents around the silicon atom can promote finer selection of perspective compound for further investigations.

## Figures and Tables

**Table 1:** Theoretically calculated lipophilicity clog *P*, IC_50_ or % inhibition values for lipoxygenase (LOX) and % carrageenin mice paw edema inhibition (CPE%) of trimethylsiloxylalkyl thiazole derivatives **12**, **14**, **16–22**. ND denotes nondetermined.

Compound	CPE%^(a)^	clog *P* ^(b)^	IC_50_ LOX

**12**	24**	3.880	9.1%
			(0.1 mmol)
**14**	35.5*	2.260	0.1 mmol
	(0.013 mmol/kg)		
**16**	47.6*	5.540	0.47 mmol
**17**	ND	1.700	0.35 mmol
**18**	ND	5.666	66.7%
			(0.1 mmol)
**19**	ND	3.939	0.01 mmol
**20**	38.3*	13.470	40.8%
			(0.1 mmol)
**21**	57.2*	3.765	ND
**22**	55.0*	4.399^(c)^	0.46 mmol
	(0.01 mmol/kg)		
Indomethacin	57.2	—	—

^(a)^ dose 0.2 mmol/kgbw.

^(b)^ theoretically calculated values using the clog *P* program from Biobyte.

^(c)^ calculated for the base.

*P < .05.

**P < .01.

**Table 2:** *In vitro* cytotoxicity against various cell lines and ability of intracellular NO generation caused by thiazolyl-(**6**), 4-(*p*-methoxyphenyl)thiazolyl-(4-hydroxypiperidyl)acetamide (**8**), and their silyl ethers **17** and **19**.

Compound	HT-1080			MG-22A			NIH 3T3	

	LC_50_ ^(a)^	LC_50_ ^(a)^	NO^(b)^	LC_50_ ^(a)^	LC_50_ ^(a)^	NO^(b)^	LC_50_ ^(a)^	LD_50,_
	CV	MTT	CV	CV	MTT	CV	NR	mg/kg

**6**	—^(c)^	—^(c)^	4	—^(c)^	> 100	6	—^(c)^	> 2000
**8**	> 100	100	8	17	16	75	185	1110
**17**	—^(c)^	—^(c)^	11	—^(c)^	—^(c)^	23	937	2132
**19**	100	100	27	21	13	122	72	839

^(a)^Concentration (*μ*g/mL) providing 50% cell killing effect (CV, MTT, and NR coloration).

^(b)^ No generation (CV coloration), determined according to [23].

^(c)^ No cytotoxic effect.
